# Prevalence of iron deficiency on ICU discharge and its relation with fatigue: a multicenter prospective study

**DOI:** 10.1186/s13054-014-0542-9

**Published:** 2014-09-30

**Authors:** Sigismond Lasocki, Nicolas Chudeau, Thibaut Papet, Deborah Tartiere, Antoine Roquilly, Laurence Carlier, Olivier Mimoz, Philippe Seguin, Yannick Malledant, Karim Asehnoune, Jean François Hamel, for the AtlanREA group

**Affiliations:** Réanimation Chirurgicale Centre Hospitalier Universitaire, Angers, France; Réanimation Chirurgicale Centre Hospitalier Universitaire, Poitiers, France; Réanimation Chirurgicale Centre Hospitalier Universitaire, Rennes, France; Réanimation Chirurgicale Centre Hospitalier Universitaire, Nantes, France; Centre de Recherche Clinique, Centre Hospitalier Universitaire, Angers, France; LUNAM Université, Université d’Angers, CHU d’Angers, Pole d’Anesthésie-Réanimation Chirurgicale, 4 rue Larrey, 49933 Angers, Cedex 9 France

## Abstract

**Introduction:**

Prevalence of iron deficiency (ID) at intensive care (ICU) admission is around 25 to 40%. Blood losses are important during ICU stay, leading to iron losses, but prevalence of ID at ICU discharge is unknown. ID has been associated with fatigue and muscular weakness, and may thus impair post-ICU rehabilitation. This study assessed ID prevalence at ICU discharge, day 28 (D28) and six months (M6) after and its relation with fatigue.

**Methods:**

We conducted this prospective, multicenter observational study at four University hospitals ICUs. Anemic (hemoglobin (Hb) less than 13 g/dL in male and less than 12 g/dL in female) critically ill adult patients hospitalized for at least five days had an iron profile taken at discharge, D28 and M6. ID was defined as ferritin less than 100 ng/L or less than 300 ng/L together with a transferrin saturation less than 20%. Fatigue was assessed by numerical scale and the Multidimensional Fatigue Inventory-20 questionnaire at D28 and M6 and muscular weakness by a hand grip test at ICU discharge.

**Results:**

Among 107 patients (men 77%, median (IQR) age 63 (48 to 73) years) who had a complete iron profile at ICU discharge, 9 (8.4%) had ID. At ICU discharge, their hemoglobin concentration (9.5 (87.7 to 10.3) versus 10.2 (92.2 to 11.7) g/dL, *P* =0.09), hand grip strength (52.5 (30 to 65) versus 49.5 (15.5 to 67.7)% of normal value, *P* =0.61) and visual analog scale fatigue scale (57 (40 to 80) versus 60 (47.5 to 80)/100, *P* =0.82) were not different from non-ID patients. At D28 (*n* =80 patients) and M6 (*n* =78 patients), ID prevalence increased (to 25 and 35% respectively) while anemia prevalence decreased (from 100% to 80 and 25% respectively, *P* <0.0001). ID was associated with increased fatigue at D28, after adjustment for main confounding factors, including anemia (regression coefficient (95%CI), 3.19 (0.74 to 5.64), *P* =0.012). At M6, this association disappeared.

**Conclusions:**

The prevalence of ID increases from 8% at discharge to 35% six months after prolonged ICU stay (more than five days). ID was associated with increased fatigue, independently of anemia, at D28.

## Introduction

Anemia is a frequent disorder in critically ill patients [1,2] and iron deficiency (ID) is known as the first cause of anemia worldwide [3]. However, ID has rarely been investigated in critically ill patients [4-6]. Our knowledge of iron metabolism was recently improved with the discovery of hepcidin [6]. There is, therefore, increasing interest in iron metabolism in critically ill patients [6].

Available studies have investigated ID in critically ill patients mainly on intensive care unit (ICU) admission [7-14]. In these studies, the prevalence of ID was relatively high, seen in 25% to 40% of the patients. In addition, blood loss (and, therefore, iron loss) is known to be significant during an ICU stay. Indeed, the mean daily volume of blood sampling is estimated from 27 to 40 ml/day [1,14,15] and the median volume of daily blood loss (estimated according to hematocrit variation) during an ICU stay has been evaluated at approximately 128 ml/day in anemic patients [14], which could represent a direct iron loss as high as 64 mg/day. In addition, because of the inflammatory response usually observed in critically ill patients, hepcidin levels are expected to be increased, leading to an impairment of dietary iron absorption (thereby impairing the physiological pathway for ID correction) [6]. One could therefore expect high ID prevalence on ICU discharge. However, to the best of our knowledge, there are no data available.

ID is a cause of anemia, but it is also responsible for fatigue and muscular weakness (with or without anemia), which can impair a patient's recovery. For example, increased fatigue has been reported after cardiac surgery in patients with ID, compared with non-ID patients [16]. Importantly, this link between ID and fatigue appears to be independent of anemia in animal studies [17,18] and in clinical reports [19]. Correction of ID after an ICU stay can also be prolonged since physiological iron uptake is quite low (approximately 1 to 2 mg/day) vis-à-vis the estimated iron deficit. One could therefore speculate that ID is frequent in critically ill patients and persists over time after ICU discharge. It could also be responsible for fatigue following ICU discharge.

The main objective of this study was to assess the prevalence of ID on ICU discharge and after (until six months after discharge) and to assess the relation between iron deficiency and fatigue.

## Material and methods

This prospective multicenter observational study was approved by the ethics committee of Angers University Hospital (approval N° 2011-28). All adult patients hospitalized for at least five days in one of the four participating surgical ICUs of the AtlanREA Group were included in the screening process (see [20] for participating center details). An informational letter was given after an oral explanation. All of the patients gave their informed consent. Patients with anemia defined by World Health Organization criteria (that is, hemoglobin (Hb) less than 12 g/dL in women or less than 13 g/dl in men) were approached on ICU discharge to give their informed consent. Patients who were unable to answer questions or who had a known iron metabolism disease were excluded. Other exclusion criteria were: chronic anemia (hemoglobin ≤10 g/dL for more than three months), current chemotherapy and estimated survival of fewer than 28 days. The guidelines for blood transfusion were based on French guidelines. Hemoglobin triggers were 8 g/dl in the case of multiple comorbidities and 7 g/dl for all other patients.

### Data collection

Data derived from medical charts were collected for all consenting patients, including age, gender, type of admission (postoperative, unplanned surgical or medical), Simplified Acute Physiology Score II (SAPS II) and Sequential Organ Failure Assessment (SOFA) for the first 24 hours following ICU admission and body mass index. Significant comorbidities were also recorded, such as history of gastroduodenal ulcer, cancer and alcoholism. Factors that could interfere with iron metabolism, nutritional state or with neuromuscular weakness were recorded: iron administration, blood transfusion, corticosteroids, vasopressor use, parenteral nutrition, renal replacement therapy and length of mechanical ventilation. ICU length of stay and major complications, such as hemorrhage, tracheotomy, ICU-acquired infection or need for emergency surgery, were also collected.

### Biological variables

The results of the iron profile routinely obtained on the day of ICU discharge were collected. Normal ranges for biological data are shown in Table 1. All hematological variables were obtained using an automated analyzer (Sysmex®, Villepinte, France). Serum iron was determined by the colorimetric method. Transferrin was determined by nephelometry. Ferritin was determined by chemiluminescent microparticle immunoassay with Architect i2000®, Abbot (Abbot, Rungis, France). Transferrin saturation was calculated as serum iron/total iron binding capacity x 100. The investigators who assessed fatigue and muscular strength (see below) were blinded to iron status.

**Table 1:** **Hematological and iron parameters on ICU admission and discharge according to iron deficiency status on ICU discharge and on D28 and at M6**

**Parameters**	***Normal ranges***	**On ICU admission**			**On ICU discharge**		
		**ID (number = 9)**	**Non-ID (number = 98)**	***P***	**ID (number = 9)**	**Non-ID (number = 98)**	***P***
Red blood cells (10^12^/L)	*4.5 to 5.8*	4.0 ± 0.6	3.7 ± 2.6	*0.15*	3.2 ± 0.6	3.7 ± 2.6	*0.21*
Hb (g/dL)	*13.5 to 17.5*	12.3 ± 1.7	11.2 ± 2.4	*0.12*	9.3 ± 1.5	10.1 ± 1.3	*0.08*
Hematocrit (%)	*40 to 50*	37.0 ± 4.8	33.5 ± 6.9	*0.09*	28 ± 5	34 ± 27	*0.09*
MCV (f/L)	*82 to 98*	91.7 ± 3.9	90.2 ± 8.5	*0.58*	89.3 ± 3.7	90 ± 3.8	*0.38*
MCHC (g/dL)	*32 to 36.5*	32.9 ± 1.9	33.1 ± 1.5	*0.51*	32.8 ± 1.2	32.8 ± 1.3	*0.44*
Reticulocyte count (10^9^/L)	*20 to 100*		37 ± 13		51 ± 35	60 ± 34	*0.67*
Serum iron (μmol/L)	*12 to 30*				6.0 ± 4.1	6.1 ± 3.4	*0.80*
Ferritin (μg/L)	*100 to 350*				196 (174 to 208)	699 (497 to 1008)	*0.0001*
Transferrin (g/L)	*1.6 to 3.2*				1.8 ± 0.5	1.6 ± 0.6	*0.19*
TSAT (%)	*20 to 40*				9 (9 to 13)	14 (10 to 19)	*0.09*

Data are presented as mean ± SD or medians (Q1 to Q3). Hb, hemoglobin; ID, iron deficiency; MCHC, mean corpuscular hemoglobin concentration; MCV, mean corpuscular volume; non-ID, no iron deficiency; TSAT, transferrin saturation. Values in italic are p values.

### Definitions of iron deficiency

Iron deficiency was defined as a serum ferritin concentration of less than 100 μg/L or as a transferrin saturation of less than 20% together with a serum ferritin concentration of less than 300 μg/L, according to the definition used in a recent interventional study [21] and by cardiologists [22,23].

### Outcome and fatigue evaluation

On ICU discharge, fatigue was assessed using a visual analog scale (VAS) with a result ranging from 0 (‘not tired’) to 100 (‘exhausted’) and by a numerical scale (patients were asked to grade their fatigue from 0 (‘not tired’) to 10 (‘exhausted’) (scores were then multiplied by 10). Hand grip tests were performed to assess muscular strength: patients were asked to squeeze a dynamometer with as much force as possible three times with their dominant hand and three times with their non-dominant hand. The best observed value was recorded. The observed value was compared with normal values according to age and gender [24]. The percentage of strength reduction was calculated using normal values according to age and gender as follows: % strength reduction = (theoretical value - measured value)/ theoretical value [24].

Patients were then followed up on day 28 (D28) and 6 months (M6) after ICU discharge. At these time points, fatigue was assessed by the Multidimensional Fatigue Inventory score (MFI-20) and by the numerical scale of fatigue. MFI-20 is a 20-item self-reporting score designed and validated to measure fatigue [25]. MFI-20 measures fatigue using five dimensions: general fatigue, physical fatigue, mental fatigue, reduced motivation and reduced activity. High MFI scores indicate high degrees of fatigue. However, we used the French validated version of the score in which only four dimensions are described (general fatigue (score ranges from 0 to 45), mental fatigue (0 to 30), reduced activity (0 to 15) and reduced motivation (0 to 10)) [26].

Finally, patients were asked to test their blood count, ferritin and transferrin saturation levels at independent laboratories on D28 and at M6. Medical prescriptions were mailed to the patients one week before the scheduled date (for patients no longer in the hospital), and patients were reminded of the need for blood panels during the phone call for assessment of fatigue.

### Statistical analysis

Data were analyzed using Stata version 12.1 (StataCorp, College Station, Texas, USA). Data are presented as medians with inter-quartile ranges (first to third quartile) or mean ± standard deviation (SD) for continuous variables and as a percentage for categorical variables.

Owing to insufficient data available to develop a satisfactory working hypothesis, an arbitrary sample size of one hundred patients was selected with an expected prevalence of ID of approximately 30% to 40%.

For the analysis, patients were separated into two groups: ID and no ID (non-ID), at the different time points evaluated (that is, ICU discharge, D28 and M6).

The primary end point was the comparison of the different fatigue scales and the strength of patients with and without ID on ICU discharge using the Wilcoxon test.

The fatigue of ID and non-ID patients was also compared on D28 and at M6. Linear regression models were created to evaluate the association between ID and fatigue on D28 and at M6. Various covariables were added to the model based on clinical judgment (ID, age, gender, SOFA, length of ICU stay, Hb on ICU discharge, type of admission, anemia, catecholamine, transfusion). The covariables retained in the model were then chosen according to the Akaike criteria. Continuous covariates included in the linear model were dichotomized when their effect could not be considered as linear. Finally, the model was validated by normalization of the residuals. The covariates finally included were presence of ID, age (dichotomized as < or ≥60 years), gender, SOFA (dichotomized as < or ≥7, which was the median SOFA score), length of ICU stay (<or ≥10 days, which was also the median length of stay), low Hb (that is, <10 g/dl).

We also created a mixed longitudinal model to evaluate the probability of ID and anemia over time after ICU discharge (with time as a fixed variable and patients as a random variable). *P* <0.05 was considered statistically significant.

## Results

**Figure 1:**
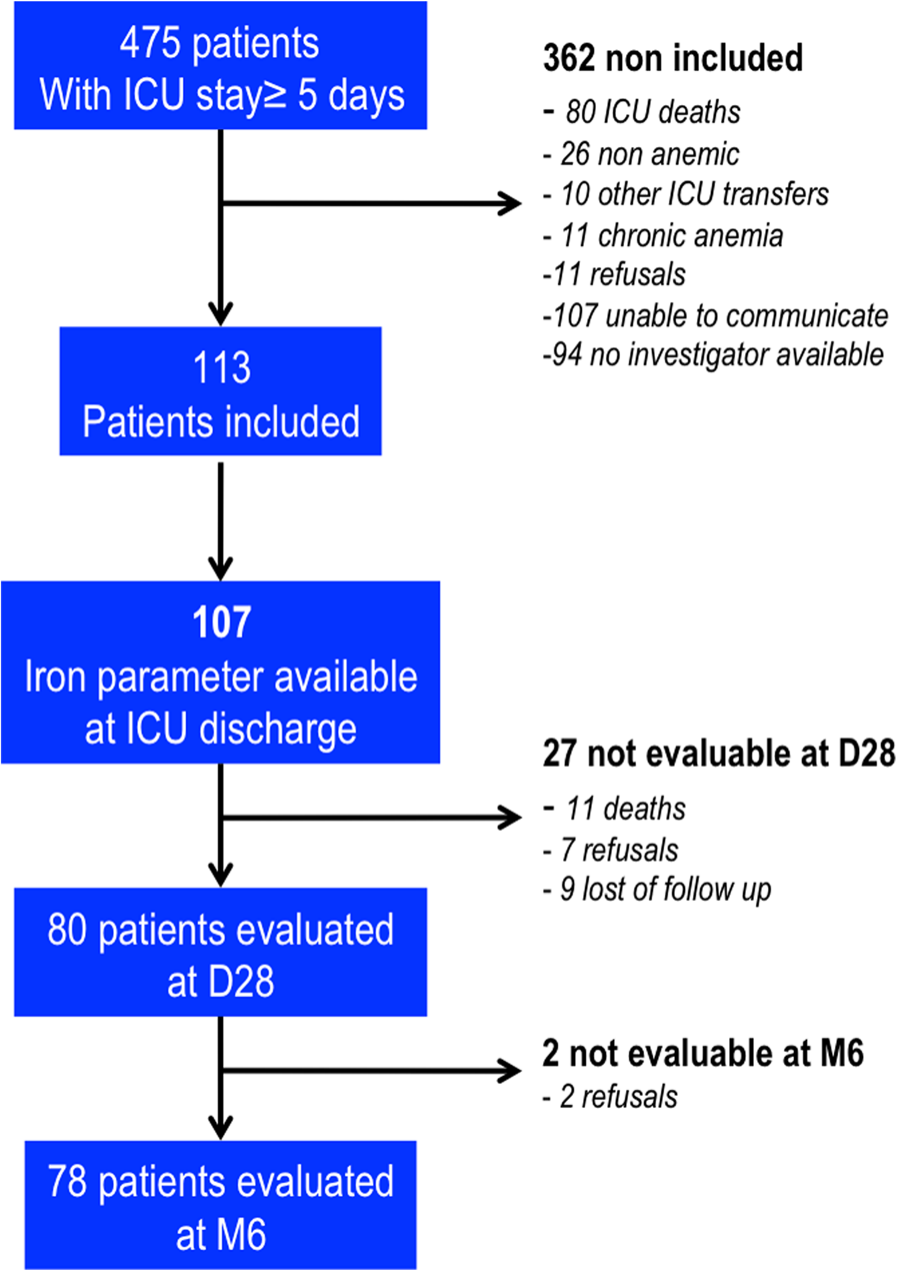
**Study flow chart.** ICU: intensive care unit; D28: 28 days after discharge; M6: 6 months after discharge.

During the study period, 475 patients admitted to the participating ICUs were eligible. Finally, 113 were included in the study and 107 had an iron profile fully available for ID diagnosis on ICU discharge. We were able to follow up 80 (75%) patients on D28 and 78 (73%) at M6 (see Figure 1 for the study flowchart). The demographic and clinical characteristics of the ID and non-ID patients were not different (Table 2). Median Hb concentration on ICU admission was 11.1 (9.3 to 12.9) g/dL. During their ICU stay, 29 (26%) patients had one or more hemorrhagic event and 60 (54%) required a median of 2 (0 to 4) units of packed red blood cell transfusions, without a difference between ID and non-ID patients.

**Table 2:** **Baseline characteristics according to discharge iron deficiency status**

**Characteristics**	**ID (number = 9)**	**Non-ID (number = 98)**	***P***
Age (years)	64 ± 18	59 ± 18	*0.45*
Body mass index (kg/m^2^)	29.7 ± 9.3	26.9 ± 6.9	*0.50*
SAPS II	52 ± 25	45 ± 18	*0.59*
SOFA	9 ± 5	7 ± 4	*0.39*
ICU length of stay (days)	11 ± 9	15 ± 12	*0.39*
Men	6 (67%)	77 (79%)	*0.42*
Type of admission			*0.9*
- unplanned surgical	6 (67%)	61 (62%)	
- scheduled surgery	1 (11%)	16 (16%)	
- medical	2 (22%)	21 (21%)	
Trauma	3 (33%)	20 (20%)	*0.2*
Hemorrhage	2 (22%)	26 (27%)	*1*
Mechanical ventilation	8 (89%)	90 (92%)	*0.56*
Renal replacement therapy	2 (22%)	19 (19%)	*1*
ICU-acquired infection	2 (22%)	39 (40%)	*0.48*
Iron supplementation	2 (22%)	2 (2%)	*0.035*
Red blood cell transfusion	3 (33%)	55 (56%)	*0.30*
Vasopressors	8 (89%)	64 (65%)	*0.27*

Data are presented as means ± SD or number (%).Hemorrhage was defined as a loss of 3 or more g/dl of Hb and/or the need for 3 or more PRBC units in fewer than 6 hours, together with a clinical source of bleeding. Iron supplementation is the use of any iron preparation (either intravenous or oral). ICU: intensive care unit; ID: iron deficiency; non-ID: no iron deficiency; PBRC: packed red blood cells; SAPS II: Simplified Acute Physiology Score II; SOFA: Sequential Organ Failure Assessment. Values in italic are p values.

### Prevalence of iron deficiency and anemia on discharge, D28 and M6

**Figure 2:**
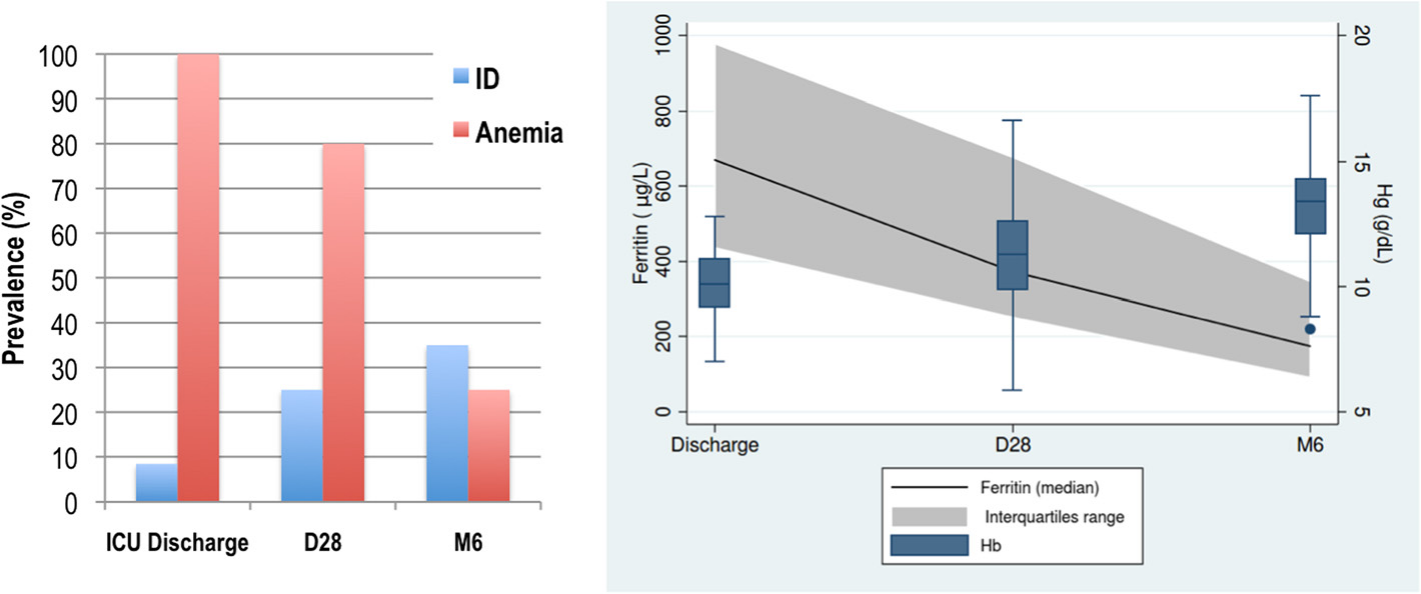
**Evolution of anemia and iron stores after ICU discharge.** The left panel represents the prevalence of anemia and iron deficiency after ICU discharge, showing an increasing ID prevalence together with a decreasing anemia prevalence. The right panel shows the evolution of ferritin and hemoglobin levels after ICU discharge. Ferritin levels (expressed as median and inter-quartile ranges) decreased from ICU discharge until M6, whereas Hb levels (medians (Q1 to Q3)) increased. D28, 28 days after discharge; ID: iron deficiency; M6, 6 months after discharge.

On ICU discharge, nine (8.4%) patients had ID with lower ferritin levels than the non-ID patients, but both groups had similar Hb concentrations (Tables 1 and 3). It should be noted that transferrin saturation remained low in both groups, indicating low iron availability in critical care patients. Interestingly, this prevalence of ID increased with time (*P* <0.0001) together with a decrease in anemia prevalence (*P* <0.0001). Twenty (25%) patients had ID on D28, and 27 (35%) at M6. We observed an increase in Hb levels from ICU discharge up to six months together with a continuous decrease in ferritin levels. However, 80% of the patients were still anemic on D28 and 25% at M6 (Figure 2).

**Table 3:** **Iron indices and Hb values on D28 and at M6**

	***Normal ranges***	**On D28**			**At M6**		
		**ID (number = 20)**	**Non-ID (number = 60)**	***P***	**ID (number = 27)**	**Non-ID (number = 51)**	***P***
Hb (g/dL)	*13.5 to 17.5*	11.5 (10.4 to 11.9)	11.3 (9.85 to 12.35)	0.26	13.6 (12 to 14.4)]	13.4 (12.2 to 14.1)	0.63
Ferritin (μg/L)	*100 to 350*	207 (98 to 269)	522 (346 to 816)	<0.0001	238 (167 to 407)	78(56 to 96)	<0.0001
TSAT (%)	*20 to 40*	14.5 (11 to 18.5)		0.002	26.8 (23 to 34)	16 (11.7 to 24)	<0.0001

### Muscular weakness and fatigue on ICU discharge

Hand grip strength was decreased on ICU discharge: median hand grip test was 44 pounds (IQR: 26 to 63) and median percentage of strength reduction was 51% (28% to 67%) depending on age and gender. Eleven (10%) patients had a strength reduction of more than 80%. However, this decrease was similar in ID and non-ID patients (40% ±27% versus 45% ±33% for ID and non-ID patients, *P* =0.27).

Fatigue assessed by a VAS and by a numerical scale of fatigue was high (55 (35 to 75)/100 and 50 (40 to 80)/100, respectively). We found a significant correlation between VAS and numerical scale of fatigue (R^2^ = 0.73 (95%CI; 0.62 to 0.81), *P* <0.0001). Only 26 (23%) patients had a VAS ≤35/100. ID and non-ID patient fatigue was similar (mean VAS 51 ± 35 versus 57 ± 24, for ID and non-ID patients, respectively, *P* =0.81).

### Outcome and fatigue on D28 and at M6

**Figure 3:**
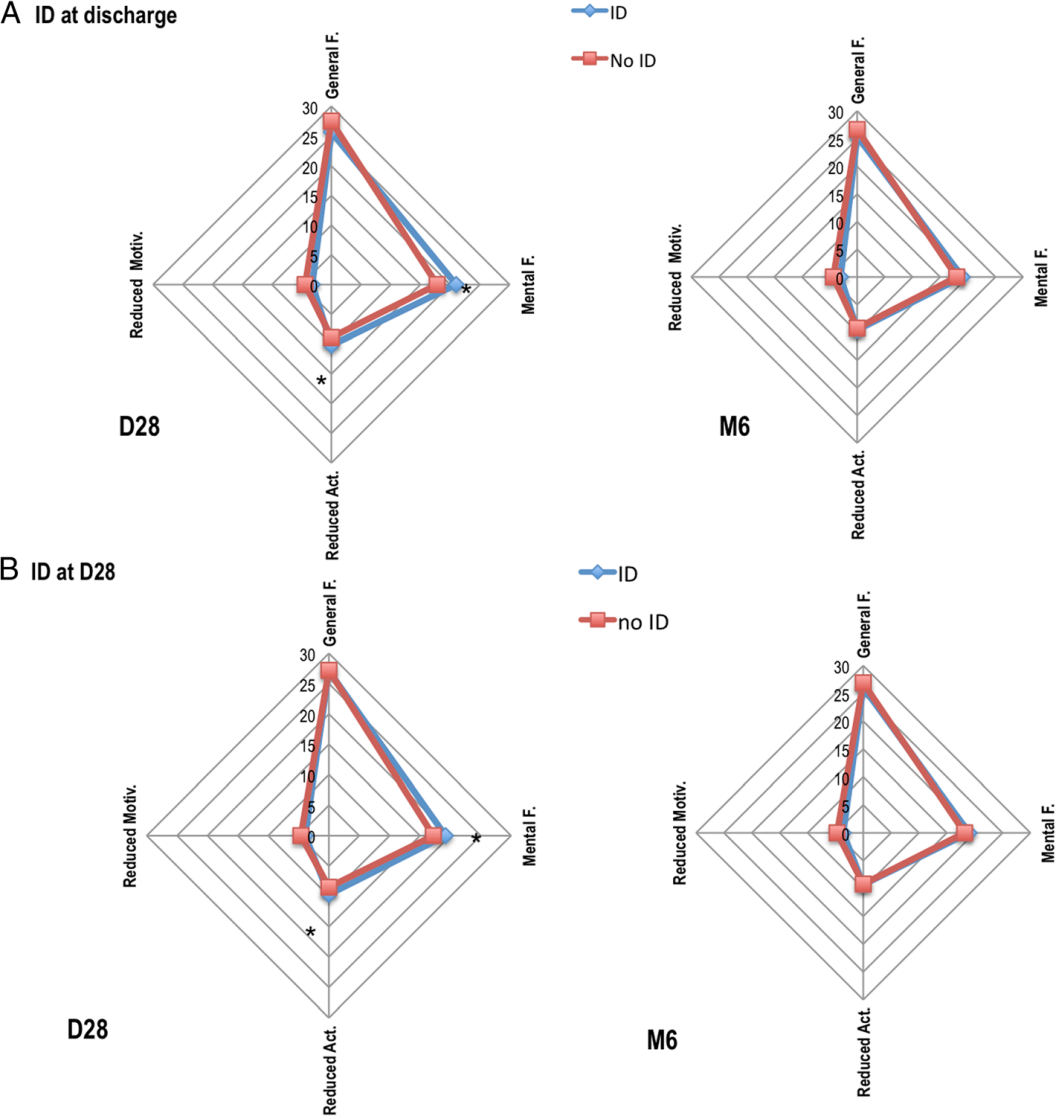
**MFI-20 scores in patients with ID on ICU discharge (Panel A) and on D28 (Panel B).** There is an association between ID, both on discharge and at D28, and higher mental fatigue and reduced activity scores. Data presented are means of each fatigue dimension. *P <0.05. D28, day 28; ID, iron deficiency; MFI-20, multidimensional fatigue inventory-20 items.

On D28, only 31 (30%) patients were alive and at home. There was a similar proportion between ID and non-ID patients (*P* =0.72). Ten (9%) patients, including one ID patient, died before D28. Two patients refused to respond to the questionnaires at six months (78 patients evaluated). On D28, two ID patients were lost to follow-up (among a total of 27 patients lost), and three (among 29) at M6. Patients with ID on ICU discharge had higher fatigue assessed by the MFI-20 at D28, with increased mental fatigue and reduced activity scores. Patients with ID on D28 also had higher MFI-20 fatigue scores in mental fatigue and reduced activity dimensions on D28 (Figure 3). At M6, ID was not associated with increased fatigue. We created multivariate linear regression models to study the link between each sub-dimension of the MFI-20 questionnaire on D28 and ID status, age, Hb and SOFA score, gender and ICU length of stay (LOS). ID was associated with mental fatigue on D28 and with reduced activity (Table 4). Age over 60 was associated with less mental fatigue, whereas an Hb of less than 10 g/dl was associated with increased general fatigue. At six months, these associations were not present. Evaluation of fatigue using the numerical scale showed no difference on D28 and at M6.

**Table 4:** **Multivariate analysis of factors associated with MFI-20 fatigue dimensions on D28**

***MFI-20 fatigue dimensions***	**Regression Coefficient (95% CI)**	***P***
**Mental fatigue**		
ID	3.19 (0.74; 5.64)	0.012
Age (>60 years)	-1.81 (-3.49; -0.13)	0.035
Hb (<10 g/dl)	-0.11 (-1.7; 1.48)	*0.89*
SOFA (>7)	1.62 (-0.12; 3.36)	*0.068*
Gender (male)	0.95 (-1.01; 2.92)	*0.34*
ICU LOS (>10 days)	-0.48 (-2.05; 1.10)	*0.55*
**General fatigue**		
ID	-2.12 (-4.41; 0.16)	*0.068*
Age (>60 years)	0.35 (-1.22; 1.92)	*0.66*
Hb (<10 g/dl)	1.82 (0.36; 3.29)	0.016
SOFA (>7)	0.86 (-0.75; 2.47)	*0.29*
Gender (male)	0.07 (-1.69; 1.83)	*0.94*
ICU LOS (>10 days)	1.08 (-0.37; 2.53)	*0.14*
**Reduced activity**		
ID	1.24 (-0.11; 2.59)	*0.07*
Age (>60 years)	-0.21 (-1.15; 0.72)	*0.65*
Hb (<10 g/dl)	-0.36 (-1.22; 0.51)	*0.41*
SOFA (>7)	0.57 (-0.38; 1.52)	*0.23*
Gender (male)	0.61 (-0.42; 1.65)	*0.24*
ICU LOS (>10 days)	0.58 (-0.27; 1.44)	*0.18*
**Reduced motivation**		
ID	-1.25 (-3.06; 0.55)	*0.17*
Age (>60 years)	-0.28 (-1.51; 0.96)	*0.66*
Hb (<10 g/dl)	0.38 (-0.81; 1.49)	*0.56*
SOFA (>7)	-0.03 (-1.29; 1.24)	*0.97*
Gender (male)	0.35 (-1.04; 1.73)	*0.62*
ICU LOS (>10 days)	-0.39 (-1.53; 0.75)	*0.49*

A linear regression model was created including: ID on ICU discharge, age (<or ≥60 years), gender (male), SOFA (<or ≥7), ICU LOS (<or ≥10 days), low Hb (Hb_D28_ < 10 g/dl) (model 1). Data are presented as OR with 95% confidence interval. Hb: hemoglobin; ICU: intensive care unit; ID: iron deficiency; LOS: length of stay;.MFI: multidimensioal fatigue inventory; SOFA: Sequential Organ Failure Assessment score. Values in italic are p values.

## Discussion

In this prospective observational study, we found that the prevalence of ID on ICU discharge was approximately 10% and increased after discharge to 35% at six months. On D28, ID was associated with increased fatigue (mental fatigue and reduced activity) but not with Hb levels.

To our knowledge, this is the first study investigating the prevalence of ID on ICU discharge. There are few data available concerning ID on ICU admission. Some studies have assessed ID on admission and found the prevalence between 9% and 38% [7-14]. These studies used different ID definitions. Bellamy *et al*. found a prevalence of 35% using the percentage of hypochromic red cells [7] and Fernandez *et al*., a prevalence of 37% using the reticulocyte Hb content [8]. In a previous study, assessing ID using different biomarkers (that is, soluble transferrin receptor, zinc protoporphyrin, transferrin saturation and ferritin) and a consensus of three experts, we found an ID prevalence of 10% on study inclusion and 26% during the ICU stay [9]. In the present study, the prevalence of ID is relatively low. Indeed, because of the blood losses regularly observed in critically ill patients (approximately 40 ml/day [15]), one could have expected a higher prevalence of ID. In our study, 26% of the patients suffered from hemorrhage and 54% required packed red blood cell transfusions during their ICU stays – suggesting significant blood losses. However, ID diagnosis is difficult in the context of inflammation [5,27]. Indeed, on ICU discharge, we observed very high ferritin concentrations in our cohort. There is no consensus to define ID in critically ill patients [5,27]. Thus, we choose to use an ID definition that has already been used in an intervention study [21] and that is commonly accepted in cardiac patients [22,23]. However, we could have used more recent biological markers that have been proposed for ID diagnosis in the presence of inflammation [28]. The most reliable test appears to be the percentage of hypochromic red cells, a value of more than 10% (normal: less than 2.5%) being indicative of a functional iron deficiency [28-30]. Reticulocyte Hb content, with a value less than 29 pg, has also been proposed [8,28,31]. Unfortunately, these two parameters are no longer usable in the case of blood transfusion, which concerns more than half of our patients. Soluble transferrin receptor is also proposed as an ID marker in the presence of inflammation [32,33]. Elevated soluble transferrin receptor reflects high bone marrow erythropoietic activity in the presence of low iron supply. However, there is no reference value and it was routinely available only at two centers out of four. Given these uncertainties, new biological markers should probably be developed and hepcidin could be one of them [9]. Hepcidin is a negative regulator of intestinal iron absorption and iron recycling by macrophages. Its synthesis is induced by inflammation and it plays a key role in the anemia of inflammation [6]. Plasma hepcidin levels are strongly repressed by ID and following stimulation of erythropoiesis, even in the presence of inflammation [34].

Even if the reported prevalence of ID on discharge is relatively low, we report for the first time much higher ID prevalence after discharge (at one and six months), with almost one-third of the patients suffering from ID at M6. In a previous study, out of 19 critical care anemic patients followed up for six months, Bateman *et al*. observed relatively low ferritin levels at six months (76 (24 to 179) μg/L) [35], in accordance with the high proportion of ID we report. Interestingly, the prevalence of ID increased after ICU discharge together with an increase in Hb levels, leading to the appearance of ID without anemia. This was also observed in the Bateman study [35]. This could be interpreted as iron being mobilized from stores to the circulating Hb. Indeed, iron absorption may not be sufficiently increased owing to persistent inflammation with elevated hepcidin levels. This could also explain the persistence of anemia in some critically ill patients [35].

We also report that fatigue was very frequent on ICU discharge and persisted on D28. Less than a quarter of our critically ill patients had a VAS of fatigue <35/100. On D28, fatigue was still significant as suggested by high MFI-20 scores and a low proportion of patients had returned home. Despite these results, we were unable to find any association between ID and fatigue on ICU discharge. There were probably too many confounding factors for fatigue on ICU discharge, such as anemia, ID, neuromuscular weakness, muscular edema and so on. Importantly, fatigue persisted on D28 and was associated with ID, independently of Hb. The relation between ID and fatigue or muscular weakness has been widely studied [36]. In cardiac surgery, for example, preoperative ID is associated with a significant increase in the physical fatigue dimension of the MFI-20 score seven days after surgery [16]. In a cohort of 28,000 women, ID was associated with fatigue and decreased general health scores [37]. Recent data have demonstrated the benefit of ID treatment on fatigue, for example, in patients with chronic heart failure even in the absence of anemia [21]. In non-anemic women, treatment of ID also improves fatigue scores compared with placebo [19]. Surprisingly, this relation between fatigue and ID was not confirmed at six months. This could be due to the definition of ID used. Indeed, ferritin concentration is dependent on the level of iron stores but also on the degree of inflammation. At D28, a ferritin concentration less than 100 μg/l is probably indicative of lower iron stores than at M6 when inflammation is expected to be lower and, thus, ferritin too. However, because we did not assess inflammation, this is only hypothetical. It is also possible that the association between ID and fatigue was observed by chance on D28.

Treatment of ID in critically ill patients may be proposed to compensate for anemia correction and to improve patient fatigue. Until recently, the potential benefit of iron was considered as being counterbalanced by its potential harm, including oxidative stress induction and infectious disease risk. Indeed, one experimental study showed increased mortality associated with high-dose iron treatment [38]. However, using therapeutic doses of iron, we found no sign of toxicity in a mouse model of critical care anemia [39] and one study of oral iron treatment in the critically ill reported a reduction in transfusion risk with no increased side effects [11]. However, further explorations are needed to confirm this hypothesis.

Our study has several limitations. First, despite a total number of 113 patients included in the study, iron profiles were available in only 107 patients. This is due to the observational design of the study. Second, we did not measure iron markers on admission or inflammatory markers, such as interleukin-6 or C-reactive protein, on day 28 and at M6. We hypothesize that high ferritin concentrations are related to a persistent inflammatory status but we cannot confirm it biologically and the diagnosis of ID (that is, the threshold of ferritin for ID) could have been modulated according to the degree of inflammation. This could be of crucial importance at M6 when inflammation is expected to be lower. As discussed above, hepcidin could have been helpful to determine ID. Third, the study is limited in that it is an observational study and, therefore, causality cannot be inferred and because of the relatively low number of patients, multiple adjustments for all comorbidities could not have been performed. Finally, we did not measure hand grip strength on D28 and at M6 owing to logistical issues.

## Conclusions

In conclusion, in our study population of surgical ICU patients, the prevalence of ID on ICU discharge was approximately 10%, underscoring the difficulty of ID diagnosis in this context. ID prevalence increases to more than one-third at six months and is associated with fatigue at one month, independently of Hb levels. Further studies are needed to evaluate the benefit of iron treatment on ICU discharge. In addition, the usefulness of other ID markers, such as hepcidin, should be investigated.

## Key messages

- The prevalence of ID was approximately 10% on ICU discharge.
- The prevalence of ID increased to 35% at six months.
- A quarter of the critically ill patients were still anemic at six months.
- ID was associated with increased fatigue one month after ICU discharge, independently of low Hb levels.

## Abbreviations

ICU: intensive care unit
ID: iron deficiency
LOS: length of stay
MFI-20: Multidimensional Fatigue Inventory score
SAPS II: Simplified Acute Physiology Score II
SOFA: Sequential Organ Failure Assessment
sTfR: soluble transferrin receptor
VAS: Visual Analog Scale

## References

[1] Vincent JL, Baron JF, Reinhart K, Gattinoni L, Thijs L, Webb A, Meier-Hellmann A, Nollet G, Peres-Bota D (2002). Anemia and blood transfusion in critically ill patients. JAMA.

[2] Corwin HL, Gettinger A, Pearl RG, Fink MP, Levy MM, Abraham E, MacIntyre NR, Shabot MM, Duh MS, Shapiro MJ (2004). The CRIT Study: anemia and blood transfusion in the critically ill–current clinical practice in the United States. Crit Care Med.

[3] **From the Centers for Disease Control and Prevention. Iron deficiency-United States, 1999-2000.** *JAMA* 2002, **288:**2114–2116. 12425310

[4] Piagnerelli M, Vincent JL (2004). Role of iron in anaemic critically ill patients: it's time to investigate!. Crit Care.

[5] Pieracci FM, Barie PS (2006). Diagnosis and management of iron-related anemias in critical illness. Crit Care Med.

[6] Lasocki S, Longrois D, Montravers P, Beaumont C (2011). Hepcidin and anemia of the critically ill patient. Anesthesiology.

[7] Bellamy MC, Gedney JA (1998). Unrecognised iron deficiency in critical illness. Lancet.

[8] Fernandez R, Tubau I, Masip J, Munoz L, Roig I, Artigas A (2010). Low reticulocyte hemoglobin content is associated with a higher blood transfusion rate in critically ill patients: a cohort study. Anesthesiology.

[9] Lasocki S, Baron G, Driss F, Westerman M, Puy H, Boutron I, Beaumont C, Montravers P (2010). Diagnostic accuracy of serum hepcidin for iron deficiency in critically ill patients with anemia. Intensive Care Med.

[10] Munoz M, Romero A, Morales M, Campos A, Garcia-Erce JA, Ramirez G (2005). Iron metabolism, inflammation and anemia in critically ill patients. A cross-sectional study. Nutr Hosp.

[11] Pieracci FM, Henderson P, Rodney JR, Holena DN, Genisca A, Ip I, Benkert S, Hydo LJ, Eachempati SR, Shou J, Barie PS (2009). Randomized, double-blind, placebo-controlled trial of effects of enteral iron supplementation on anemia and risk of infection during surgical critical illness. Surg Infect (Larchmt).

[12] Rodriguez RM, Corwin HL, Gettinger A, Corwin MJ, Gubler D, Pearl RG (2001). Nutritional deficiencies and blunted erythropoietin response as causes of the anemia of critical illness. J Crit Care.

[13] van Iperen CE, Gaillard CA, Kraaijenhagen RJ, Braam BG, Marx JJ, van de Wiel A (2000). Response of erythropoiesis and iron metabolism to recombinant human erythropoietin in intensive care unit patients. Crit Care Med.

[14] von Ahsen N, Muller C, Serke S, Frei U, Eckardt KU (1999). Important role of nondiagnostic blood loss and blunted erythropoietic response in the anemia of medical intensive care patients. Crit Care Med.

[15] Lyon AW, Chin AC, Slotsve GA, Lyon ME (2013). Simulation of repetitive diagnostic blood loss and onset of iatrogenic anemia in critical care patients with a mathematical model. Comput Biol Med.

[16] Piednoir P, Allou N, Driss F, Longrois D, Philip I, Beaumont C, Montravers P, Lasocki S (2011). Preoperative iron deficiency increases transfusion requirements and fatigue in cardiac surgery patients: a prospective observational study. Eur J Anaesthesiol.

[17] Finch CA, Miller LR, Inamdar AR, Person R, Seiler K, Mackler B (1976). Iron deficiency in the rat. Physiological and biochemical studies of muscle dysfunction. J Clin Invest.

[18] Willis WT, Gohil K, Brooks GA, Dallman PR (1990). Iron deficiency: improved exercise performance within 15 hours of iron treatment in rats. J Nutr.

[19] Krayenbuehl PA, Battegay E, Breymann C, Furrer J, Schulthess G (2011). Intravenous iron for the treatment of fatigue in nonanemic, premenopausal women with low serum ferritin concentration. Blood.

[20] **AtlanRea.** [www.atlanrea.org]

[21] Anker SD, Comin Colet J, Filippatos G, Willenheimer R, Dickstein K, Drexler H, Luscher TF, Bart B, Banasiak W, Niegowska J, Kirwan BA, Mori C, von Eisenhart Rothe B, Pocock SJ, Poole-Wilson PA, Ponikowski P, FAIR-HF Trial Investigators (2009). Ferric carboxymaltose in patients with heart failure and iron deficiency. N Engl J Med.

[22] Jankowska EA, Rozentryt P, Witkowska A, Nowak J, Hartmann O, Ponikowska B, Borodulin-Nadzieja L, Banasiak W, Polonski L, Filippatos G, McMurray JJ, Anker SD, Ponikowski P (2010). Iron deficiency: an ominous sign in patients with systolic chronic heart failure. Eur Heart J.

[23] Okonko DO, Mandal AK, Missouris CG, Poole-Wilson PA (2011). Disordered iron homeostasis in chronic heart failure: prevalence, predictors, and relation to anemia, exercise capacity, and survival. J Am Coll Cardiol.

[24] Mathiowetz V, Kashman N, Volland G, Weber K, Dowe M, Rogers S (1985). Grip and pinch strength: normative data for adults. Arch Phys Med Rehabil.

[25] Smets EM, Garssen B, Bonke B, De Haes JC (1995). The Multidimensional Fatigue Inventory (MFI) psychometric qualities of an instrument to assess fatigue. J Psychosom Res.

[26] Gentile S, Delaroziere JC, Favre F, Sambuc R, San Marco JL (2003). Validation of the French 'multidimensional fatigue inventory' (MFI 20). Eur J Cancer Care (Engl).

[27] Heming N, Montravers P, Lasocki S (2011). Iron deficiency in critically ill patients: highlighting the role of hepcidin. Crit Care.

[28] Thomas DW, Hinchliffe RF, Briggs C, Macdougall IC, Littlewood T, Cavill I (2013). Guideline for the laboratory diagnosis of functional iron deficiency. Br J Haematol.

[29] Urrechaga E, Borque L, Escanero JF (2009). Potential utility of the new Sysmex XE 5000 red blood cell extended parameters in the study of disorders of iron metabolism. Clin Chem Lab Med.

[30] Urrechaga E (2009). The new mature red cell parameter, low haemoglobin density of the Beckman-Coulter LH750: clinical utility in the diagnosis of iron deficiency. Int J Lab Hematol.

[31] Thomas L, Franck S, Messinger M, Linssen J, Thome M, Thomas C (2005). Reticulocyte hemoglobin measurement–comparison of two methods in the diagnosis of iron-restricted erythropoiesis. Clin Chem Lab Med.

[32] Thomas C, Kirschbaum A, Boehm D, Thomas L (2006). The diagnostic plot: a concept for identifying different states of iron deficiency and monitoring the response to epoetin therapy. Med Oncol.

[33] Weiss G, Goodnough LT (2005). Anemia of chronic disease. N Engl J Med.

[34] Lasocki S, Millot S, Andrieu V, Letteron P, Pilard N, Muzeau F, Thibaudeau O, Montravers P, Beaumont C (2008). Phlebotomies or erythropoietin injections allow mobilization of iron stores in a mouse model mimicking intensive care anemia. Crit Care Med.

[35] Bateman AP, McArdle F, Walsh TS (2009). Time course of anemia during six months follow up following intensive care discharge and factors associated with impaired recovery of erythropoiesis. Crit Care Med.

[36] Haas JD, Brownlie T (2001). Iron deficiency and reduced work capacity: a critical review of the research to determine a causal relationship. J Nutr.

[37] Patterson AJ, Brown WJ, Powers JR, Roberts DC (2000). Iron deficiency, general health and fatigue: results from the Australian Longitudinal Study on Women's Health. Qual Life Res.

[38] Javadi P, Buchman TG, Stromberg PE, Husain KD, Dunne WM, Woolsey CA, Turnbull IR, Hotchkiss RS, Karl IE, Coopersmith CM (2004). High-dose exogenous iron following cecal ligation and puncture increases mortality rate in mice and is associated with an increase in gut epithelial and splenic apoptosis. Crit Care Med.

[39] Heming N, Letteron P, Driss F, Millot S, El Benna J, Tourret J, Denamur E, Montravers P, Beaumont C, Lasocki S (2012). Efficacy and toxicity of intravenous iron in a mouse model of critical care anemia. Crit Care Med.

